# Drawings of THINGS: A large-scale drawing dataset of 1854 object concepts

**DOI:** 10.3758/s13428-025-02887-w

**Published:** 2026-01-30

**Authors:** Kushin Mukherjee, Holly Huey, Laura M. Stoinski, Martin N. Hebart, Judith E. Fan, Wilma A. Bainbridge

**Affiliations:** 1 Department of Psychology, University of Wisconsin-Madison, Madison, WI USA; 2 Wisconsin Institute for Discovery, University of Wisconsin-Madison, Madison, WI USA; 3 Max Planck Institute for Human Cognitive and Brain Sciences, Leipzig, Germany; 4 Department of Psychology, University of California, San Diego, CA USA; 5 University of Leipzig, Leipzig, Germany; 6 International Max Planck Research School on Cognitive NeuroImaging, Leipzig, Germany; 7 Department of Medicine, Justus Liebig University, Giessen, Germany; 8 Center for Mind, Brain and Behavior, Universities of Marburg, Giessen, and Darmstadt, Marburg, Germany; 9 Department of Psychology, Stanford University, Stanford, CA USA; 10 Department of Psychology, University of Chicago, Chicago, IL USA; 11 Neuroscience Institute, University of Chicago, Chicago, IL USA

**Keywords:** Drawing, Big data, Visual concepts, Object recognition, Memory, Typicality

## Abstract

The development of large datasets of natural images has galvanized progress in psychology, neuroscience, and computer science. Notably, the THINGS database constitutes a collective effort towards understanding of human visual knowledge by accumulating rich data on a shared set of visual object concepts across several studies. In this paper, we introduce Drawing of THINGS ( **DoT** ), a novel dataset of 28,627 human drawings of 1854 diverse object concepts, sampled systematically from concrete picturable and nameable nouns in the American English language, mirroring the structure of the THINGS image database. In addition to data on drawings’ stroke history, we further collected fine-grained recognition data for each drawing, along with metadata on participant demographics, drawing ability, and mental imagery. We characterize people’s ability to communicate and recognize semantic information encoded in drawings and compare this ability to their ability to recognize real-world images of the same visual objects. We also explore the relationship between drawing understanding and the memorability and typicality of the objects contained in THINGS. In sum, we envision **DoT** as a powerful tool that builds on the THINGS database to advance understanding of how humans express knowledge about visual concepts.

**Supplementary Information:**

The online version contains supplementary material available at 10.3758/s13428-025-02887-w.

## Introduction

A central goal in the cognitive sciences is to understand how semantic knowledge is organized in the mind and brain. *Visual* semantic knowledge (Thompson-Schill et al., 1999; Warrington & Shallice, 1984) in particular is implicated in object recognition (DiCarlo et al., 2012; De Lange et al., 2018; Gauthier & Tarr, 2016; Huth et al., 2012; Rogers et al., 2003), the encoding of objects in memory (Rogers et al., 2004; Konkle et al., 2010; Cunningham & Wolfe, 2014), and organizing perceived similarity among objects (Mur et al., 2013; Charest et al., 2014; Tversky, 1977; Rosch, 1975; Muttenthaler et al., 2022; Hebart et al., 2020). Recent years have seen important advances in the elucidation of the representational structure of visual semantic knowledge – how concepts are situated with respect to each other and the features that shape this organization (Hebart et al., 2020; Fu et al., 2023; Caplette & Turk-Browne, 2024; Mur et al., 2013). This has been facilitated by two key advances: (1) the development of large image datasets that are representative of the object concepts that people are familiar with (Hebart et al., 2019, 2023; Mehrer et al., 2021; Allen et al., 2022) and (2) the dense annotation and augmentation of the items within these datasets with behavioral and neural measurements (Stoinski et al., 2023; Grootswagers et al., 2022; Kramer et al., 2023; Hansen & Hebart, 2022; Vanasse et al., 2022). Experimental results based on such datasets are valuable insofar as conclusions derived from them are not restricted to a handful of experimenter-curated stimuli and are much more likely to generalize broadly. The use and development of these datasets have also contributed to a more cohesive body of research with methods and data that are interoperable across studies, a strategy that has been fruitful in other fields, especially computer science (Deng et al., 2009; Schuhmann et al., 2022; Everingham et al., 2010; Sharma et al., 2018; Lin et al., 2014).

Here we harness the study of *drawing production and recognition* at scale to advance understanding of visual semantic knowledge. While open-ended verbal production, such as generating feature lists for concepts, has been central to investigations of semantic cognition for many decades (McRae et al., 2005; Devereux et al., 2014; De Deyne et al., 2008), there has been a rapid recent development of methods for using visual production to investigate the content and organization of conceptual knowledge (Mukherjee & Rogers, 2024; Mukherjee et al., 2019; Long et al., 2024; Fan et al., 2023). Drawing tasks have been used in studies of perception (Biederman & Ju, 1988; Sayim & Cavanagh, 2011; Yang & Fan, 2021), development (Long et al., 2024; Dillon, 2021), learning (Fan et al., 2018; Chamberlain et al., 2021), memory (Bainbridge et al., 2021, 2019; Megla et al., 2025; Bozeat et al., 2003), and others. The importance and utility of using line drawing-based pictorial representations of objects as a tool for understanding visual and semantic cognition can be seen in the widespread adoption of even early datasets such as the one curated by Snodgrass et al. (1980) in the study of human (Thompson-Schill et al., 1997; Patterson et al., 2007; Alvarez & Cavanagh, 2004; Singer et al., 2023) and machine (Kubilius et al., 2016; Singer et al., 2022; Mukherjee et al., 2024) vision. Due to their abstract nature and divergences in low-level visual features relative to real-world objects, drawings have also served as key test items for the cognitive benchmarking of artificial vision and learning systems (Singer et al., 2022; Mukherjee et al., 2024; Mukherjee & Rogers, 2024; Boutin et al., 2023; Wang et al., 2021; Lake et al., 2015). Thus, drawings provide ways to probe human visual knowledge using open-ended responses that complement the closed-set response space used in standard recognition and discrimination tasks. This open-ended nature of drawing is important because it can provide insight into the contents of human mental representation in trial-efficient ways. For example, Bainbridge et al. (2019) used a drawing-based approach to ask participants to reproduce visual scenes from memory. The use of drawing as a medium allowed the researchers to detect when participants failed to reproduce specific objects or when they mis-remembered where objects were located within the scene. Drawing tasks have also been used to characterize the enrichment of visual concept knowledge throughout middle childhood (Long et al., 2024), and to characterize what parts of objects people deem relevant to include in a drawing depending on their communicative goals (Fan et al., 2020; Mukherjee et al., 2019; Huey et al., 2023).

Several drawing datasets already exist that were developed using web-based tools (De Leeuw, 2015; Bainbridge, 2022) and crowdworking platforms, such as Amazon Mechanical Turk (AMT). Sheepmarket (Koblin, 2009), a dataset of over 10,000 drawings of sheep collected from such a set of crowd workers, was an early proof of concept that reasonably high-quality drawing data can be collected through this medium. Later, Eitz et al. (2012) developed the TU-Berlin dataset representing 250 classes, but with over 20,000 drawings in the dataset drawn by non-experts on Amazon Mechanical Turk (AMT), increasing the overall semantic diversity and scale of drawing datasets. A similar approach of collecting crowdsourced drawings was adopted by Sangkloy et al. (2016), who collected over 75,000 unique drawings of 125 categories to build the Sketchy dataset. A unique aspect of this latter dataset was that each drawing was based on a real-world photograph, allowing for comparisons of human and machine visual recognition of the common objects across modalities. Google’s Quick! Draw game (Jongejan et al., 2017), an online game where people could make drawings and have an AI system guess a label for it, led to the development of a dataset of over 50 million sketches of 345 categories, the first dataset to allow for training data-intensive deep learning models (Ha & Eck, 2017). Long et al. (2024) introduced a drawing dataset of over 37,000 drawings of 48 object classes made by children between the ages of 2 and 10, allowing for investigations into changes in the structure of visual concepts across development. These datasets have been instrumental in galvanizing progress towards understanding how humans flexibly deploy their visual knowledge to create visual representations that abstract and distill from their real-world counterparts, yet nevertheless often suffice for effective communication (Hawkins et al., 2023).

**Fig. 1:**
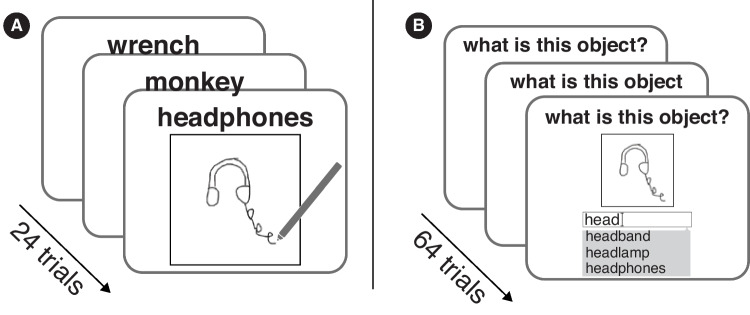
**A** In Experiment 1, each participant made drawings of 24 unique objects. **B** In Experiment 2, separate participants provided up to five labels for each of 64 drawings sampled from the drawings made in Experiment 1

While these prior approaches have laid critical theoretical and methodological groundwork, they are nevertheless limited in several ways. First, despite the largest of these datasets being on the scale of several million individual drawings, the diversity of the object classes in Quick Draw! falls short of the range of visual concepts represented in modern investigations of visual concepts (Hebart et al., 2023) nor do they capture the *kinds* of object categories that align with human visual experience (Mehrer et al., 2021). Second, it is difficult to relate findings on drawings of the visual objects in these datasets to findings about the same objects more generally due to the absence of commensurate large-scale data either on the drawing or real-world image side. A path forward is to integrate drawing dataset collection efforts within a larger ecosystem of research findings grounded in shared test items.

Here, we introduce *Drawings of THINGS* ( **DoT** ), a dataset of over 28,000 drawings of 1854 visual objects that adopts such an integrative approach. **DoT** differentiates itself from prior datasets most saliently along the axis of semantic diversity. A critical feature of this dataset lies in the variety of semantic categories represented, leveraging the richness of THINGS (Hebart et al., 2019, 2023), a database of 26,107 images of 1854 object concepts with psychologically relevant metadata. Importantly, the objects in THINGS were carefully sampled from an extensive set of concrete nouns in American English, ensuring a selection that is both broad and distinctive, while focusing on the most common and easily identifiable concrete concepts (e.g., dog, sweater, acorn). Accompanied by extensive metadata and object property ratings (Stoinski et al., 2023; Hebart et al., 2023), THINGS supports the precise selection of stimuli, conditions, and control variables. Additionally, the growing number of publicly available datasets and studies utilizing the THINGS concepts (Hebart et al., 2023; Grootswagers et al., 2022; Gifford et al., 2022; Kramer et al., 2023; Mukherjee et al., 2024; Dobs et al., 2022; Hebart et al., 2020; Benchetrit et al., 2023; Papale et al., 2025) facilitates comparisons of findings across different disciplines, methods, and species. As a result, **DoT** constitutes a new semantically diverse drawing dataset that supports asking a multitude of questions about visual knowledge, while dovetailing with the rich THINGS database ecosystem that allows us to connect each drawing to corresponding object photographs, metadata, and neural data.

In addition to the drawings themselves, we also collected fine-grained human recognizability data on each drawing and on photographs of the same objects in the THINGS database. For each drawing, we also collected stroke-order information that could be used as a proxy for what information is prioritized first or last in drawing a given object. This form of temporal stroke data has been critical in building computational models of human concept learning (Lake et al., 2015). We also include data on each sketcher’s demographics, drawing ability, imageability, and educational background. We demonstrate how this dataset can be used to test hypotheses about visual semantic knowledge and its relationship to other aspects of visual cognition.

Lastly, we make available all our data for the community to develop their own studies and test a larger array of hypotheses.

## Methods

### Experiment 1: Drawing production study

Our first goal was to collect a large-scale dataset of drawings of the 1854 objects present in the THINGS database. Similar to recent studies employing drawing-based methods (Yang & Fan, 2021; Bainbridge et al., 2019; Hawkins et al., 2023; Fan et al., 2020; Megla et al., 2025; Mukherjee et al., 2024), we used Prolific, an online crowdsourcing platform, to collect multiple drawings of each of the THINGS objects.

#### Participants

A total of 1316 participants were recruited on the online experimental platform Prolific. Participants were allowed to complete multiple sessions contingent on maintaining satisfactory ratings. We excluded data from two participants due to technical errors in data collection, leaving a sample size of 1314 participants (735 male, 511 female, 68 other/did not wish to say^1^; $$M_{age}$$= 37.04). All participants provided informed consent in accordance with the University of California San Diego Institutional Review Board (IRB) and were compensated at $15/hour.

#### Procedure

**Fig. 2:**
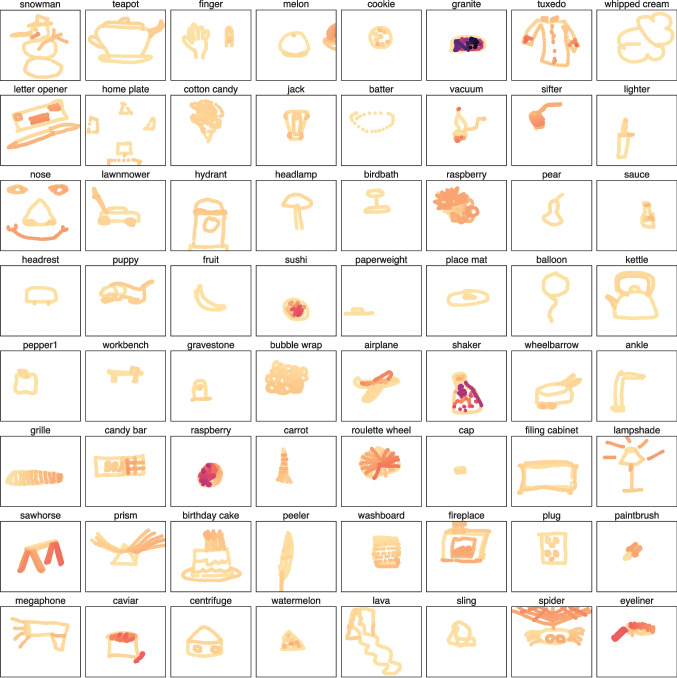
Sample drawings from **DoT** rendered stroke-by-stroke. *Darker strokes* indicate those made later during the drawing process

The experiment was designed using jsPsych version 7 (De Leeuw, 2015) using a variant of its sketchpad plugin. On each trial of the experiment, participants were presented with a blank 550px $\times$ 550px canvas surrounded by a black border of size 2px and an object word above the canvas. Object words were obtained from the names of the 1854 objects contained within the THINGS database, which capture most concrete nameable objects in human experience (e.g., apple, chinchilla, laptop). In cases where the label was ambiguous (such as ‘bat’ the animal or ‘bat’ the sporting good), additional disambiguating text, obtained from the THINGS+ dataset (Stoinski et al., 2023), was presented in parentheses (e.g., bat (animal)). We appended such disambiguating text to 204 out of the 1854 concepts. The participants’ task was to use their touch pad, mouse, or any other cursor device to make a drawing of the prompted object such that a naive viewer would be able to guess the object’s label from looking at the drawing (Fig. 1A). Participants could make their drawings only in a single color (black) and were given the option to undo their most recent stroke, redo the stroke in case they accidentally undid an action, and also to clear the entire canvas if they wanted to restart their drawing. The undo, redo, and clear actions were implemented as buttons near the bottom of the canvas. We only allowed participants to participate through a desktop or laptop computer, asking them to retry if they logged into our task using a phone or tablet. Participants were free to spend as much time on each drawing as they wanted and had to at least make one stroke to be allowed to proceed to the next trial. There was a button to ‘submit’ the drawing and continue on to the next trial when they were done with each drawing. In addition to saving the image of the drawings made by participants, we also saved the order of strokes made by participants (Fig. 2). After each drawing trial, participants were asked whether they knew what the prompted object looked like in real life, which they could answer with either ‘yes’ or ’no’. This was to flag potential low-quality drawings that were a result of the sketcher not being familiar with the queried object. Each participant completed 24 drawing trials, with the median time taken to complete the experiment being 21 min. Before completing the experiment, participants completed a questionnaire where they reported their demographic information, including age, gender, ethnicity, state of residence (optional), and level of education. They were also asked to answer seven-point Likert scale questions on self-reported drawing skill and mental imagery capabilities ("*When you try to form a mental picture, it is usually:*", where the endpoints of the scale were ‘no image’ and ‘very clear’).

#### Filtering for high-quality drawings

We collected a total of 29,876 drawings with an average of 16.2 drawings per THINGS object (*SD* = 4.69), with the smallest number of drawings collected for an object being 12 and the largest being 41. In order to filter out drawings that were inappropriate, not relevant to the prompted object label, or blank canvases, we had a team of six internal annotators rate the validity of each drawing given the prompted object. Not all annotators saw each drawing but each drawing was shown to at least three annotators. The annotators were asked to indicate if a given drawing was ‘valid’ or ‘invalid’ according to the following specific criteria – invalid drawings were those that consisted of (1) illegible scribbles, (2) text written out in the form of a drawing, or (3) offensive imagery. Since there is some degree of subjectivity to these criteria, we collected at least three validation ratings for each of the 29,876 drawings. We chose to only include drawings in our final dataset that were rated as ‘valid’ by the majority of the annotators, i.e., at least two out of three. This criterion allowed for screening out of obviously bad drawings while remaining conservative in terms of data exclusion. This led us to remove 1249 drawings (4.18% of the total number of drawings), which yielded a final dataset of 28,627 drawings.

### Experiment 2: Drawing recognition study

After collecting the dataset of drawings, we ran a second study with the aim of characterizing patterns of recognition behavior for these drawings. To this end, we conducted an experiment where a new cohort of participants had to provide object labels for each of the drawings in our dataset.

#### Participants

A total 1578 participants were recruited on Prolific to complete the drawing recognition study. Participants were allowed to complete multiple sessions contingent on maintaining satisfactory ratings. Thirteen participants either did not complete even a single recognition trial or faced technical difficulties during the experiment. We excluded an additional eight participants who failed to provide reasonable labels for a catch trial embedded within normal recognition trials, which depicted a drawing of a cat. Our final sample consisted of 1557 participants (744 male, 674 female, 32 other, six did not wish to say^2^; $$M_{age}$$= 39.96). All participants provided informed consent in accordance with the UC San Diego and University of Chicago IRBs. Participants were compensated at $8/hour.

#### Stimuli

The stimuli were the 28,627 drawings in our dataset that remained after exclusion of poor-quality drawings. The set of all possible labels was each of the THINGS 1854 object labels (with the appropriate disambiguating text in parentheses for the relevant objects).

#### Procedure

Participants were tasked with providing semantic labels (i.e., object names) for the drawings. The labels they could provide were restricted to objects in the THINGS database. On each trial of the experiment, participants were provided with a random drawing from Experiment 1 and text above the drawing asking ‘What is this object?’. There was an empty text box below the drawing where the participant could type an answer. As the participant typed, several candidate objects would be populated in a drop-down menu based on partial string matches to the THINGS objects. The participant had to select one of the possible drop-down labels and could not enter a label that was not in the set (Fig. 1B). Given that some drawings could be ambiguous or may evoke more than one semantic label, we allowed participants to provide up to five labels, although only one label was required to progress to the next trial. Before starting the experiment, participants completed a practice trial to familiarize themselves with the interface. All participants were shown the same researcher-made drawing of a cat during this practice trial. While we did not exclude any participants based on whether they correctly labeled this practice trial, 95.89% of the participants provided the exact label ‘cat’, and 99.73% of participants provided a label that was either ‘cat’, ‘kitten’, ‘fox’, or ‘dog’ showing that our interface was intuitive to use and participants generally understood the task.

The same drawing was presented once more during the actual experimental trials as a ‘catch trial’. Only 8 participants (0.5%) failed to pass this check. To begin the experiment, participants had to correctly answer three comprehension check questions that tested that they knew not to enter multiple labels of the same object, not to leave the response text box empty, and to answer with their best guess when they weren’t sure of the object depicted in the drawing. Each participant completed 64 recognition trials where they were never shown more than one drawing of each THINGS object. Before completing the experiment, participants completed a questionnaire where they reported their demographic information, including age, gender, ethnicity, and highest level of education. They also reported their mental imagery using the scale described in Experiment 1.

Drawings from each object were labeled by an average of 55.67 participants, with each individual drawing being labeled by an average of 3.63 participants. On average, participants provided 1.52 (*SD* = 0.57) labels per drawing with a maximum of five labels provided for a single drawing. Participants spent an average of 18.01 s per recognition trial (*SD*=2.65 s).

### Experiment 3: Image recognition study

We additionally collected a dataset of recognizability scores for all photographs included in the THINGS database (Hebart et al., 2019; Stoinski et al., 2023). Similar to the drawing recognition study, participants were instructed to select the most fitting label for the object depicted in each photograph from a drop-down menu containing the 1854 THINGS object labels. The goal of this experiment was to provide complementary recognition data on the same visual objects when depicted using photographs as opposed to line drawings.

#### Participants

A total of 13,158 participants were recruited via the crowdsourcing platform Amazon Mechanical Turk. All participants resided in the US and provided informed consent in compliance with the Ethics Committee of the Medical Faculty of Leipzig University, Germany. Participants received a small compensation for each completed HIT and could choose to participate in as many HITs as they wished.

To ensure data quality, several exclusion criteria were applied during and after data collection. Participants were flagged as potentially non-compliant if they completed five trials in under 1100 ms, or all ten trials in under 1300 ms. Additionally, participants were flagged as non-compliant if, in five or more trials, they selected one of the top drop-down options after typing only a single letter in the drop-down menu search bar. Individuals flagged as potentially non-compliant on two occasions were prevented from further participation using a custom function embedded in the experimental script. Each HIT included one easily recognizable catch image. After data collection, participants were flagged as non-compliant if they mislabeled the catch images in 50% or more of their HITs. Workers flagged as non-compliant had all their data excluded from the analysis. Following these exclusions, 8094 individuals remained (4607 female, 3395 male, 92 diverse), ranging in age from 18 to 98 years (*M* = 37.81, *SD* = 11.25). On average, participants completed 6.10 HITs (*SD* = 58.94).

#### Stimuli

Participants provided labels for all 26,107 images in the THINGS dataset (Hebart et al., 2019), along with the 1854 public domain images provided in THINGS+ (Stoinski et al., 2023).

#### Procedure

A single image recognizability experiment (human intelligence task, HIT) consisted of ten trials. In each trial, participants were presented with an image and asked to select the most appropriate label for the depicted object from a drop-down menu listing the 1854 THINGS objects (Hebart et al., 2019). Similar to the drawing recognition experiment, the labeling interface allowed participants to begin typing with the dropdown auto-suggesting labels based on partial matches. For objects with ambiguous meanings, additional context was provided in parentheses. Here, participants provided only a single label per image.

Each HIT included one easily nameable catch image in a randomly assigned trial, assuming that participants who failed to label this image likely were noncompliant in completing the experiment. As catch images, we selected 2796 unique THINGS images that were correctly named in 100% of cases during the free label generation task of the THINGS+ nameability experiment (Stoinski et al., 2023). Initially, each image was sampled 20 times. After excluding trials from noncompliant participants, we conducted a follow-up collection to fill up the number of samples per image back up to 20. As every HIT had to include one catch image, some catch images were sampled slightly more often. After data exclusion, each individual image had been labeled between 5 and 23 times (*M* = 14.70, *SD* = 2.40). Averaged across the 13 or more image examples per object, each object was labeled between 165 and 532 times (*M* = 221.71, *SD* = 41.44).

**Fig. 3:**
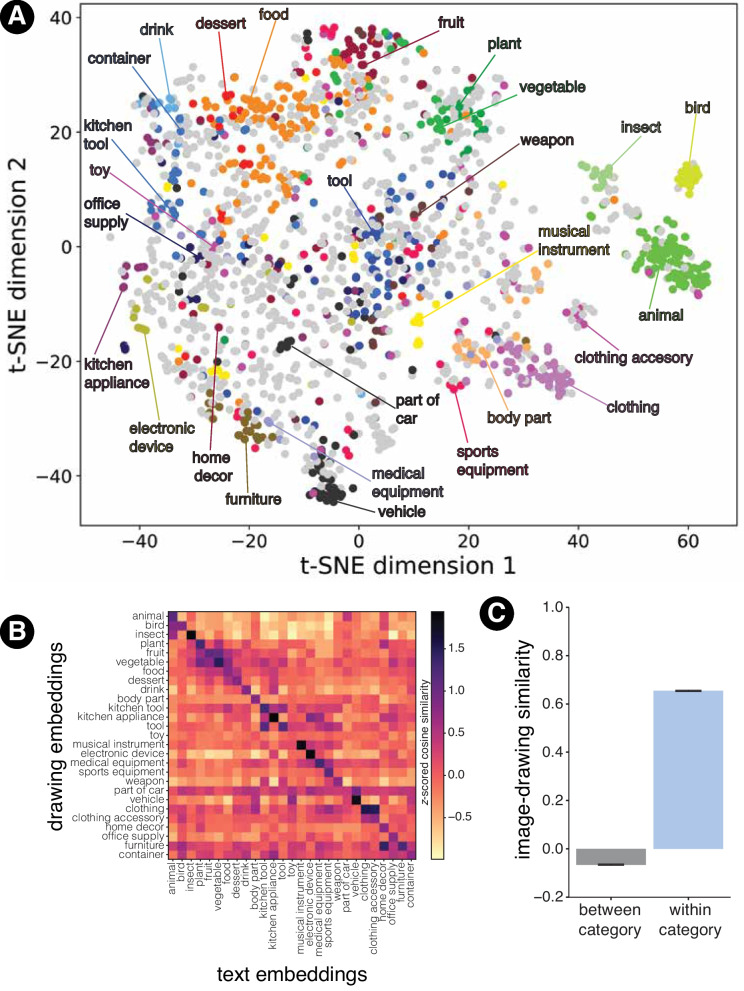
**A** t-SNE visualization of the mean SigLIP representation of drawings of each of the 1854 THINGS categories. Objects belonging to the 27 core categories are highlighted in different colors. **B** Similarity between the mean SigLIP image embeddings of drawings from the 27 core categories with each of the text embeddings for the 27 core category labels. **C** Similarity between image and drawing SigLIP embeddings when the images and drawings are from different object categories (*gray*) and from the same object category (*light blue*)

## Results

The final **DoT** dataset consists of 28,627 drawings belonging to 1854 object classes. Each drawing is associated with a 550px $\times$ 550px raster image representation of the drawing, a history of strokes used to make the drawing, which can be used to re-render the drawing stroke-by-stroke, the amount of time the participants took to complete the drawing, the mean recognizability of that drawing^3^, and whether the sketcher was familiar with the object or not. We additionally provide embeddings for each drawing obtained from SigLIP (Zhai et al., 2023), a state-of-the-art multimodal transformer model as described in the following section.

### Drawings in **DoT** are visually diverse while selectively evoking their true semantic categories

Computer vision models trained for object recognition have been shown to be good models of human vision across a variety of behavioral and neural metrics (Yamins et al., 2014; Cadieu et al., 2014; Mahner et al., 2025). Recent years have seen the emergence of alternative self-supervised training objectives such as visual contrastive learning (Konkle & Alvarez, 2022; Zhuang et al., 2021; Chen et al., 2020) and image-language contrastive learning (Radford et al., 2021), and transformer-based vision models are competitive with and even surpass convolutional neural networks at aligning with human behavior despite sharing fewer inductive biases with primate vision (Conwell et al., 2024). We leveraged such a transformer-based multimodal model SigLIP (Zhai et al., 2023), which was trained on web-scale corpora of images and text data, and computed embeddings for each of the drawing images to test the diversity of their representations. For simplicity, below, we report results on drawings belonging to the 27 superordinate ‘core’ categories identified by Hebart et al. (2019) (e.g., food, furniture, clothing). We first selected drawings of objects that belonged to the 27 categories. This resulted in a set of 13,802 drawings that we show in Fig. 3A. Each point corresponds to the position of a single object obtained by averaging the SigLIP feature vectors for all drawings of that object and then projecting the high-dimensional vector into a two-dimensional space using the t-stochastic neighbor embedding algorithm. The mean drawing-to-drawing similarity expressed by SigLIP reveals that drawings of some objects appear highly clustered (e.g., animals, birds, clothing, and weapons) while drawings of other categories appear more dispersed (e.g., tools and food). Overall, it does appear that **DoT** qualitatively spans a reasonably large degree of visual variety while organizing objects in a semantically coherent manner.

To quantify this semantic coherence and measure the extent to which the drawings serve as good candidates for the core categories they belong to (without looking at the human recognition data), we once again used SigLIP to estimate the extent to which the visual embeddings of drawings belonging to each category aligned with the text embeddings for its category label. We conducted these analyses at the level of the core categories to align closely with the analyses in Hebart et al. (2019), where the authors showed that semantic embeddings of the THINGS objects showed category selectivity for the core categories. For each drawing, we computed the dot product between its SigLIP image embedding and the SigLIP text embedding of each of the 27 core categories. This allowed us to compute the difference between image-to-label similarities between each drawing and its target category and the mean similarities to the remaining 26 categories. If this difference is positive, it constitutes a measure of how selectively that drawing evoked the target core concept relative to the others. We conducted a permutation test where we scrambled the category labels and repeated the procedure 1000 times to estimate statistical significance. Figure 3B shows the cosine similarities between the average image embedding for each category (averaged over all drawings belonging to that category) and the text embeddings for each of the category labels. Similarities were *z*-scored to put them on the same scale. We found that, for the majority of categories, their drawings were significantly selective for the true label except for ‘part of car’ ($p < 0.001$ for 26 out of 27 categories after Bonferroni corrections for multiple comparisons).

A key benefit of organizing the present drawing dataset around the THINGS database is that it allows for comparing the visual information conveyed in object drawings to naturalistic images of the same objects. To examine if the visual features underlying drawings and images are reliably similar, we extracted SigLIP features from the 26,107 naturalistic images from the THINGS database (Hebart et al., 2019). Next, we computed average object embeddings using both the image SigLIP features and the drawing SigLIP features we previously extracted. Using these averaged embeddings, we computed a 1854 $\times$ 1854 cosine similarity matrix representing image-to-drawing object similarities. To quantify if images and drawings of the same category (e.g., apple (drawing) vs. apple (image)) were reliably more similar than mismatched images and drawings (e.g., apple (drawing) vs. radio (image)), we computed the average of the diagonal of this similarity matrix ( *M* = 0.67, *SD* = 0.05; light blue bar in Fig. 3 C) and compared it to the average of the off-diagonal entries (gray bar; *M* =-0.07, *SD* = 0.05). To account for the fact that some objects are inherently similar to other objects visually, we corrected the off-diagonal entries of the matrix by subtracting from them the off-diagonal values from an image-to-image similarity matrix. Overall, the ‘within-category’ image-sketch embedding pairs were more similar than the ‘between-category’ pairs ($p < 0.001$^4^), which suggests that drawings of objects visually evoke their target object more than other objects.

Thus, using machine vision metrics, we show that **DoT** exhibits a high degree of category selectivity, thus indicating that the drawings visually evoke the core categories they are made to evoke. For the category that didn’t show high selectivity for its true labels (part of car), this is perhaps expected given that many parts of cars were closer to the label ‘vehicle’ relative to the true label. In subsequent sections, we investigate to what extent this selectivity holds true for human observers and whether they can discern semantic structure in drawings at even finer-grained levels of analysis.

### Object drawings in **DoT** vary in recognizability

Having established that **DoT** is visually diverse and that individual drawings broadly adhere to the semantic categories of the objects they depict, we turned our attention to investigating the degree to which human observers could recognize the semantic information that sketchers had encoded in their drawings. A representative measure of the semantic information within a drawing is the object label assigned to it by participants in our drawing recognition study (Experiment 2). Thus, we began by measuring how often participant-provided labels were exact matches to the true label for each drawing. We compared the object-level recognizability scores derived from drawings to recognizability scores derived from photographs of the same objects (Experiment 3) in order to characterize the extent to which drawings of objects were more or less efficient at conveying semantic information relative to real-world exemplars.

In order to measure how often the object label inferred by an observer matched the label provided to the sketcher during the production task, we first computed the proportion of guessed labels that were an exact match to the label shown to the sketcher of each drawing. We measured this at two levels — (1) by testing if the first label provided by each participant in the recognition experiment matched the true label (top-1 accuracy) and (2) by testing whether *any* of the labels provided by each participant matched the true label (top-*k* accuracy). For the image recognition experiment, we computed an accuracy metric where we allowed homonyms of the correct label to also be considered ‘correct’, assuming that they were likely selected by mistake; for example, if ‘button (clothes)’ was chosen instead of the intended label ‘button (device)’. We report results using the latter score.

We found a high degree of variability among the recognizability of drawings of objects, with the top-1 accuracy spanning the entire range of possible values (*M*=21.79%; *SD*=22.84%; max = 96.15%, min = 0%). We observed a similar trend for top-*k* accuracy with an expected slight increase in accuracy (*M*=26.50%; *SD*=24.39%; max=100%, min = 0). Moving forward, when we refer to recognizability for drawings, we are generally referring to the top-*k* metric unless otherwise specified. While drawings of objects like ‘ladder’,‘snowman’, and ‘cactus’ were almost always recognized and labeled correctly, others like those of ‘mongoose’, ‘mint’, and ‘moccasin’ failed to *ever* elicit the correct label (Fig. 5).

### Sketchers’ self-reported drawing skills and mental imagery influences their drawings’ recognizability

**Fig. 4:**
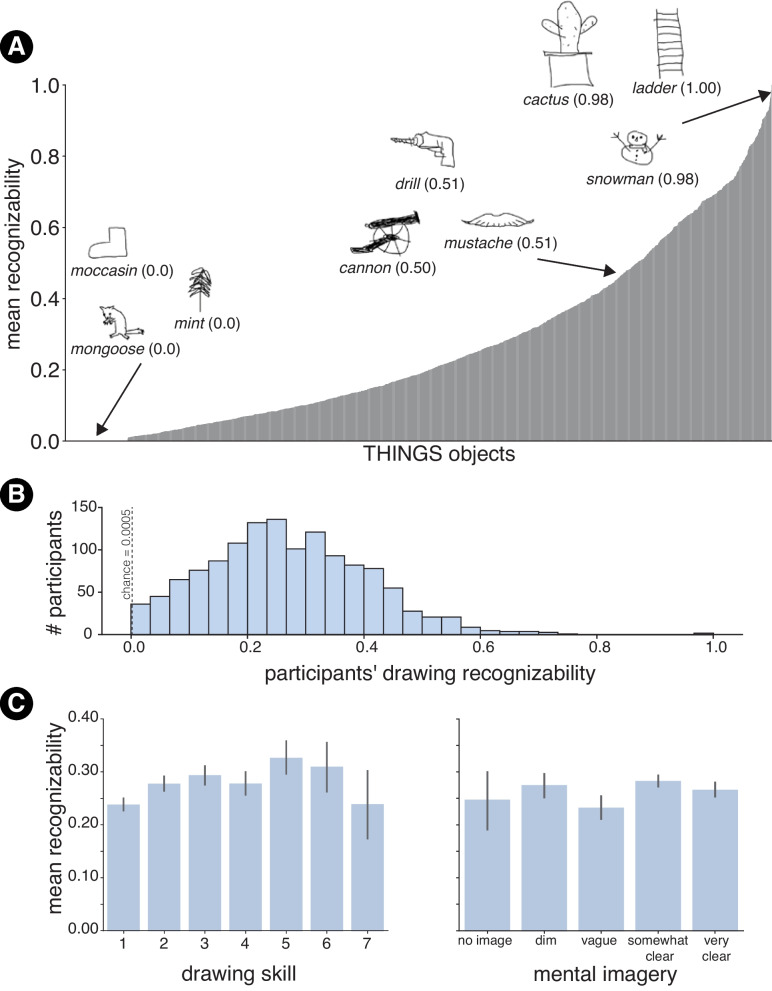
**A** Distribution of mean drawing recognizability across all 1854 THINGS concepts sorted from least recognizable to most recognizable. *Numbers in parentheses* next to highlighted examples correspond to mean top-*k* accuracy for that object. **B** Distribution of Experiment 1 participants’ drawing ability measured using the mean recognizability of drawings made by them. **C** Average recognizability of participants’ drawings as a function of their self-reported drawing skills and mental imagery

Since sketchers self-reported their familiarity with each prompted object label during the drawing trials, we calculated the mean object recognizability separately for trials where they recognized the label and for those where they did not. Of the 1854 objects used in **DoT** , 1300 objects had drawings that were judged to be both familiar and unfamiliar at least once with the remaining 554 objects always judged as familiar. For the set of 1300 objects, we found that, on average, drawings of objects where the sketcher was familiar with the object label were more recognizable (*M* = 22.09%, *SD* = 21.99%) than drawings of objects where the sketcher was not familiar with the object label (*M* = 11.49%, *SD* = 22.98%) (*t*(1, 853) = 17.69, $p < 0.001$). Thus, perhaps unsurprisingly, familiarity with the prompted object led sketchers to produce more recognizable drawings. The fact that recognizability for drawings for the ‘unfamiliar’ group was not 0 indicates that some sketchers may have either misunderstood what the question was asking or that even not knowing what the object looks like in ‘real life’ did not preclude them from possessing the requisite knowledge to make a drawing that nevertheless conveyed the visual concept. More generally, the average recognizability of the drawings made by a given sketcher constitutes a measure of their drawing ability. To investigate the distribution of drawing abilities, we computed sketcher-specific mean recognizability scores by averaging over the recognizability scores for all the drawings that they produced. The distribution of scores is shown in Fig. 4 B. While there was variability in participants’ ability to convey the target object using drawings (*SD* = 13.85%), on average a sketcher made drawings that were recognizable 26.99% of the time, much higher than chance (1/1854) with 70 out of 1314 (5.32 %) sketchers having average recognizability scores over 50% and 23 sketchers (1.75%) having an average recognizability lower than chance.

To test whether a person’s drawing skill was predictive of their drawings’ recognizability, we fit a linear regression model predicting the average recognizability of sketchers’ drawings from their self-reported drawing skill ratings. We found a small effect of self-reported drawing skill on drawing recognizability ($\beta$ = 0.02, *SE* = 0.002, $p < 0.001$; model $R^2$ = 0.03), indicating that people are generally well calibrated in assessing their drawing ability and how recognizable their drawings will be. A similar analysis of the relationship between people’s self-reported mental imagery ratings and drawing recognizability did not reveal a significant relationship ($\beta$ = 0.003, *SE* = 0.004, $p = 0.43$), suggesting that one’s subjective perception of their mental imagery vividness was not a reliable predictor of how recognizable their drawings would be.

### Recognizability of object drawings is correlated with recognizability of object photographs

**Fig. 5:**
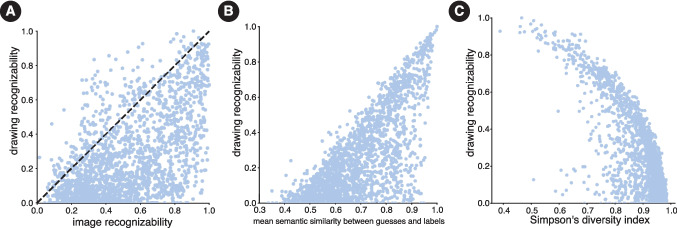
Each *point* in each of the three plots corresponds to a single THINGS object. **A** Recognizability of drawings of objects as a function of recognizability of images of the same objects. **B** Drawing recognizability as a function of the mean similarity between guessed labels and the true labels, averaged over drawings for each object. **C** Drawing recognizability as a function of label diversity

In order to evaluate whether the low recognizability scores could be attributed to the fact the drawings were made by non-expert artists and simply provided insufficient visual information to inform a correct guess, we compared the recognizability of each object as measured using drawings with scores for the same objects when assessed using images. Overall, recognition accuracy for images of objects (*M* = 52.65%, *SD*= 24.41% ) was higher than recognition accuracy for drawings of objects (*M* = 26.50%, *SD*= 24.39%) (*t*(1, 853)=39.49, $p < 0.001$). However, since there was comparable variance in recognizability for both domains, the key question was whether the objects that had low recognizability scores for drawings were the same ones that had low recognizability scores for photographs. To test this, we first estimated a noise ceiling for how reliable the rank-ordering of object recognizability was. This was done by first generating two samples of the image recognizability data by sampling 20 recognition trials for each object with replacement and computing Kendall’s $\tau$ between the rank ordering of objects in terms of their mean recognizability scores. This procedure was repeated 1,000 times using different splits of the data to estimate a mean Kendall’s $\tau$ (*M* = 0.89, *SD* = 0.002). This constitutes a reliability measure of the rank ordering of objects based on photograph recognizability and the best that we can expect for the rank ordering of object recognizability between drawings and photographs. Using this measure, we next computed a ‘noise-corrected’ rank-order correlation between objects’ drawing recognizability scores and image recognizability scores. Generally, we found that objects that were difficult to recognize as drawings were also difficult to recognize in photographs (corrected Kendall’s $\tau (1,853)$ = 0.42, $p < 0.001$) (Fig. 5 A).

However, there were several cases that diverged from this general trend. The points above the identity line in Fig. 5 A correspond to such objects that were more easily recognized as drawings than as photographs. To quantify the degree to which objects were easier to recognize as drawings relative to images, we computed a recognizability difference score by subtracting the average recognizability score for images of an object from the recognizability score for drawings of that object ($recognizability_{drawing} - recognizability_{photo}$). Objects that had a positive score for this metric were those that were easier to recognize as drawings relative to photographs.

Objects such as ‘ticktacktoe’ (0.48, 95% CI = [0.03 – 0.93]^5^), ‘reindeer’ (0.44, 95% CI = [-0.01, 0.90]), ‘palm tree’ (0.44, 95% CI = [-0.01, 0.90]), and ‘popsicle’ (0.43, 95% CI = [-0.02, 0.89]) were among the objects that had the highest drawing − image recognizability scores. Overall, 211 of the 1854 (11.38%) objects were more easily recognized as drawings relative to photographs on average. At the same time several objects were *harder* to recognize when rendered as drawings as opposed to photographs (e.g., ‘chocolate’ (-0.89, 95% CI = [-1.34, -0.44]), ‘tiger’ (-0.68, 95% CI = [-1.13, -0.23]), and ‘frog’(-0.56, 95% CI = [-1.01, -0.11])).

Thus, even when considering all guesses that the participants provided for each drawing, we found a high degree of variability in terms of how easily object drawings were recognized. We additionally found that, while the recognizability of real-world instances of objects is predictive of drawing recognizability, there exist objects that are more efficiently communicated using drawings over photographs than others. We next considered whether the incorrect labels might also contain signatures of people’s visual knowledge of the queried objects.

### Drawings vary in visual complexity

The earliest of standardized drawing datasets collected and used in the psychological sciences included, among other metadata, ratings on visual complexity (Snodgrass et al., 1980). The motivation was that the visual complexity of a depiction could impact cognitive processing, including but not limited to naming latencies, recognizability, and memorability. Recent years have seen these ideas corroborated to various degrees with the visual complexity of visual displays being linked to how engaging they are (Sun & Firestone, 2021), how easily they are recalled (Borkin et al., 2015), and their affective associations (Madan et al., 2018).

**Fig. 6:**
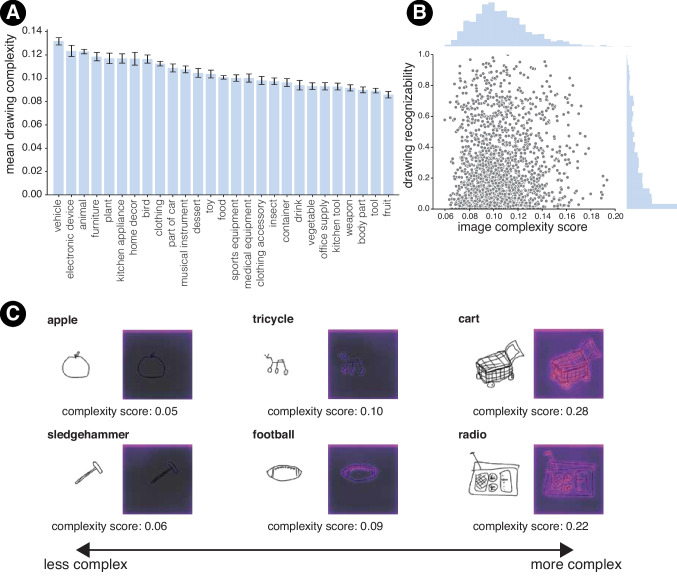
**A** Mean drawing complexity of the core 27 THINGS categories. **B** Distributions of average recognizability scores and complexity scores for drawings of each THINGS object category. **C** Examples of low, medium, and high complexity drawings, with complexity scores and saliency maps derived from ICNet (Feng et al., 2022)

A recurring issue has been how complexity should be operationalized and measured. Previous approaches have used explicit human ratings (Snodgrass et al., 1980; Madan et al., 2018; Olivia et al., 2004; Heaps & Handel, 1999; Feng et al., 2022), various instantiations of description lengths (Lewis & Frank, 2016; Sun & Firestone, 2022), and automatic algorithmic or machine-driven processes (Madan et al., 2018; Sun & Firestone, 2022; Feng et al., 2022; Shen et al., 2024; Wilder et al., 2016). Given the scale of **DoT** , we sought to use the third approach to annotate each drawing in the dataset with a visual complexity score. Specifically, we used the ICNet model proposed by Feng et al. (2022). This model consists of a two-stream convolutional neural network architecture with a specialized attention module that allows the model to take into account low-level detailed features and the overall spatial layout of an image to produce both a visual complexity score and a saliency map over the input image, highlighting the points of maximum complexity (Fig. 6C). We chose this method due to its ability to outperform a range of comparable computer vision methods for estimating human visual complexity ratings on a variety of image datasets. Using this model, we estimated complexity scores and saliency maps for each drawing in **DoT** .

We found that the drawings in **DoT** varied in their overall visual complexity (*M* = 0.11, *SD* = 0.04; min. = 0.03, max. = 0.35; Fig. 6B). Further, we found that, on average, drawings of different categories showed systematic variation in visual complexity. Figure 6A shows the average complexity scores for each of the 27 core THINGS categories. The category ‘vehicles’ showed the highest degree of visual complexity (*M* = 0.13, *SD* = 0.04), ‘fruits’ showed the least complexity (*M* = 0.09, *SD* = 0.03), and categories like ‘toys’ showed an intermediate level of complexity (*M* = 0.10 , *SD* = 0.04). To show that object category influences the drawing’s visual complexity at a more granular level, we fit a linear regression model predicting drawings’ complexity scores with a categorical predictor for object category. Compared to a reduced model that did not include a predictor for object category, we found that the full model explained significantly more variance (*F*(1853, 101266) = 29.79, $p < 0.001$), indicating that object categories are generally important for determining the complexity of the resulting drawing.

Lastly, we observed a weak but significant positive correlation between visual complexity and drawing recognizability (*r*(1852) = 0.07, $p< 0.005$), suggesting that the strokes that induce greater visual complexity might also help support the recognizability of a drawing, albeit marginally so.

### Diversity of guessed labels predicts lower drawing recognizability

The previous section established that there was variability in the degree to which drawings of objects were accurately labeled. We measured whether this diversity in labels generated by participants for drawings of objects was predictive of that object’s recognizability. The rationale was that to the extent that label diversity does *not* predict recognizability, this would suggest that some drawings systematically convey the wrong object. Alternatively, if greater label diversity was predictive of lower recognizability, then drawings might be evoking many different objects in the eyes of an observer (e.g., drawings of dogs, a four-legged animal, might be labeled as other four-legged mammals such as cows, horses, zebras, etc.).

For each drawing, we operationalized the label diversity using Simpson’s diversity index (SDI) (Simpson, 1949). Concretely, the SDI for each drawing was given by –

$$\begin{aligned} SDI = 1 - \frac{ \sum _{i} n_i (n_i-1)}{N(N-1)} \end{aligned}$$

where *N* refers to the total number of labels provided for the sketch across participants, including the optional 2nd to 5th guesses, and $n_i$ refers to the number of guesses made for the *i*th object. The more dispersed the guesses for an object are across all the possible labels (i.e., the more participants disagree on what is depicted in the drawing), the closer the SDI is to 0. Conversely, the more concentrated the guesses are around a few labels (i.e., the more participants agree on what is depicted in the drawing), the closer it is to 1. In general, we found that guesses tended to be concentrated around a few labels (SDI *M* = 0.88, *SD*= 0.09), but that there was still variability across objects (max SDI = 0.99 ; min SDI = 0.38). We found a strong negative correlation between an object’s SDI and its recognizability (Pearson’s *r*(1, 852) = -0.80, $p < 0.001$) (Fig. 5 C). This supports the notion that when observers converge on a single label for a given drawing, it is often the correct label that was shown to the sketcher during the production task. However, it leaves open the question of why drawings with high label diversity exist.

For cases where observers are generating multiple labels for drawings of a given object and still getting them ‘wrong’, there are two broad possibilities — (1) the drawing lacked the visual information to allow an observer to correctly identify the intended object and thus they responded randomly, or (2) the drawing did convey enough information for the observer to recognize it, but they failed to use the appropriate label given the semantic diversity of 1854 objects in THINGS, many of which are semantic ‘neighbors’ (e.g., kitten and cat). In order to better adjudicate between these two possibilities, in the following section, we looked beyond exact-match metrics to a semantic matching approach where we measured whether guessers produced labels that were in the correct ‘semantic neighborhood’ of the true labels.

### Label guesses semantically approximate the true label

In order to establish a graded metric for how similar different objects were, we leveraged *semantic embeddings* for each of the 1854 object categories collected by Hebart et al. (2023). These embeddings position each of the THINGS objects in a common 66-dimensional space, where the inter-item vector distance reflects the item-to-item similarity as assessed using 4.6 million human triplet odd-one-out judgments (Zheng et al., 2019; Stoinski et al., 2023; Hebart et al., 2023). Since these embeddings were computed based on similarities perceived between photographs of the THINGS objects as opposed to drawings, it was first important to validate the generalizability of these embeddings for this particular stimuli class of drawings. In order to do so, we tested whether the similarity between the true labels for drawings of a given object and observers’ guesses for drawings of that object in embedding space was predictive of the likelihood of that object being labeled correctly.

For each drawing, we computed how similar a participant’s guess was to the *true* label by computing the cosine similarity between semantic embeddings of the guess and true label. In cases where there were multiple guesses, we adopted two strategies. First, we computed the similarity between the true label and the mean embedding of the guessed labels. Overall, we found that an object’s *mean guess similarity*, that is, how similar guesses were to the true label, was predictive of its recognizability (Pearson’s *r*(1852)= 0.71, $p <0.001$) (Fig. 5 (B)). This approach would penalize a participant if one of the multiple guesses was semantically distal from the true label. So, as an alternative approach, we computed the similarity between the embedding of the true label and the *closest guess*. We found that this *best guess similarity* was slightly more predictive of an object’s recognizability (Pearson’s *r*(1852) = 0.74, $p < 0.001$). Thus, the similarity structure expressed by real-world photographs of visual objects generalizes and is able to capture how recognizable visually abstract drawings of the same objects are.

Having established that item-wise similarity between labels and guesses in semantic embedding space is predictive of whether drawings of objects are recognizable, we next sought to use these semantic embeddings to measure whether *incorrect* guesses were nevertheless semantically related to the true label. Concretely, we used the similarity of incorrect label guesses to the target label to derive such a measure of the object’s *semantic neighbor preference* (Mukherjee et al., 2024). In order to compute semantic neighbor preference (SNP) for a given object’s drawings, we first determine a rank ordering of the 1853 *off*-target labels in terms of their similarity to the true target label (based on their semantic embeddings). The higher the rank of a given object label, the closer a neighbor it is to the true object label for that drawing. In order for a drawing to have high SNP, the rank of all its off-target labels should be reasonably high (that is, they should be close to 1). We next computed the cumulative proportion of off-target labels as a function of their rank. A drawing that elicits labels neighboring the true label will have most of its assigned labels ranked highly (i.e., lower numerical ranks), causing the cumulative proportion to rise steeply toward 1. In contrast, if many assigned labels are semantically distant, their ranks will be lower (i.e., higher numerical values), leading to a more gradual increase. This rate of increase is captured by the area under the curve (AUC) of the cumulative proportion vs. label rank plot. This AUC value corresponds to the SNP metric. A SNP value closer to 1 indicates that guesses were in the same ‘semantic neighborhood’ of the true label and thus that drawing has a high semantic neighbor preference. In contrast, an SNP value close to 0.5 would indicate that the off-target labels were indeed random and that there is no semantically meaningful structure in the incorrect guesses. We computed SNP values for each object by averaging the SNP values for each drawing belonging to the object.

**Fig. 7:**
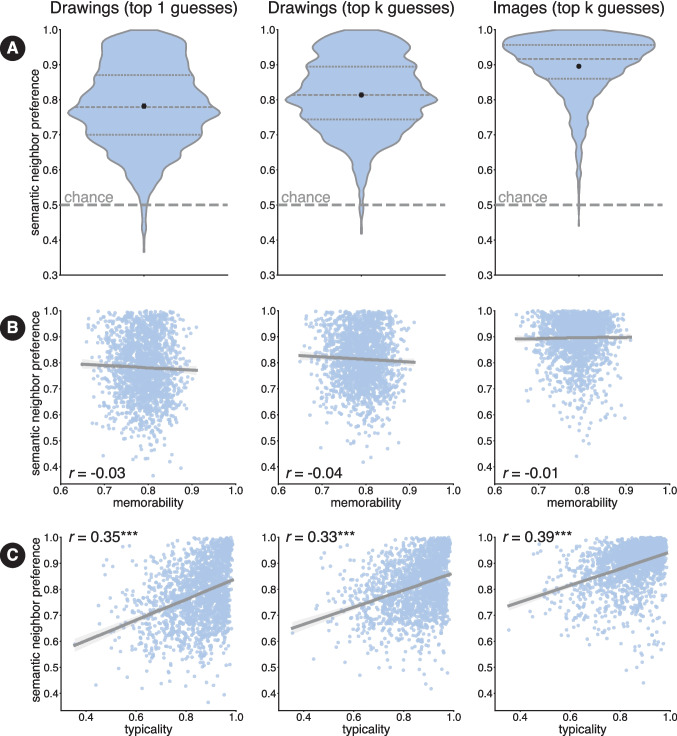
**A** Distribution densities of semantic neighbor preference scores across objects. *Dashed lines* within violin plots indicate medians and quartiles and the *black dots* indicate means. **B** Semantic neighbor preference as a function of object memorability. **C** Semantic neighbor preference as a function of object typicality. *Asterisks* indicate statistical significance: *** $p < 0.001$

We generally found that most objects had a high semantic neighbor preference (*M*=0.78, *SD*= 0.12) relative to a uniform baseline (0.5), indicating that even when participants do not provide the exact true label, their guesses are systematic and are semantically related to the true label. Even after accounting for the fact that different labels may nevertheless ‘point’ to the same underlying object, our SNP scores continued to span a considerable range (max = 0.99, min = 0.36). In addition to computing SNP based on the first guess a participant made, we also computed a ‘top-*k*’ variant in a manner similar to what we previously did for computing recognizability. In cases where a participant was incorrect and provided multiple labels, we considered the label that was closest to the true label in semantic embedding space as the guess and computed the rank of that label. Considering the ‘top-*k*’ guesses led to an overall higher SNP (*M*=0.81; *SD* = 0.10; max = 0.99, min = 0.42).

Despite this range, the vast majority of objects (1846 of 1854; except ‘bagpipe’,‘bomb’, ‘boomerang’, ‘chest’ (furniture), ‘chest’ (container), ‘football’, ‘mouse’ (device), and ‘ring’) showed an SNP value greater than 0.5, supporting the notion that generally objects’ off-target labels were semantically meaningful. We also estimated the SNP using the photograph recognizability data and found that photographs yielded even higher SNP scores (*M* = 0.90, *SD* = 0.08; max = 0.99, min = 0.44) (summarized in Fig. 7 A). The SNP scores between drawings (top-k guesses) and photographs (top-k guesses) were also moderately correlated (Pearson’s *r*(1852) = 0.37, $p < 0.001$), indicating that observers make similar patterns of off-target guesses when presented with visual object concepts in both drawing and photographic form. It is not surprising that drawings and images are not perfectly correlated in terms of SNP, since drawings, by nature, are more abstract and might thus evoke a broader variety of visual concepts relative to less abstract images.

For subsequent analyses, we utilized SNP as a measure of observers’ sensitivity to semantic information in drawings, as it reflects guesses within the semantic neighborhood of the correct label. This shift can be understood as a gradual expansion of what qualifies as an “acceptable” label. Moving from top-1 to top-*k* accuracy broadens the range of participant responses considered correct based on predefined criteria. In contrast, transitioning from accuracy to SNP represents a shift in the researcher-defined target itself, focusing on the semantic proximity of responses rather than strict correctness. The series of analyses in the present section raises the question as to whether cognitively circumscribed properties of these visual objects may determine the extent to which people are able to produce and consequently recognize drawings of them. To illustrate the potential of **DoT** as a standardized dataset that interfaces with existing large-scale resources germane to the cognitive sciences, in the following section, we present an analysis of our data that utilizes data from existing THINGS datasets.

### Typicality but not memorability of concepts yield more recognizable drawings

One overarching goal of the original THINGS dataset was to provide a representative sample of objects, covering often-ignored object classes, to help explain the factors that influence recognition and recall of these objects in naturalistic behavior (Hebart et al., 2019). Here, we explore whether two candidate cognitive factors – memorability (Bainbridge, 2019) and typicality (Rosch & Mervis, 1975; Rosch et al., 1976) – might help explain the pattern of results we find with respect to drawing and image recognition. Memorability is a temporally consistent image-specific property that is predictive of one’s ability to remember that stimulus. Sensitivity to memorability develops early in childhood (Guo & Bainbridge, 2024), is not purely determined by low-level visual features of images (Bainbridge & Rissman, 2018), and is largely independent from other top-down cognitive processes such as cognitive control (Bainbridge, 2020). While work investigating the underlying features of memorability on naturalistic images is nascent (Kramer et al., 2023), here we consider that a visual concept might inherit the memorability of its exemplars, including drawings, leading to shared memorability between photographs and drawings of the same object (Han et al., 2023). Since it has been shown that semantic properties are the most predictive of concept-level memorability (Kramer et al., 2023), to the extent that recognizing drawings of objects recruits the same features used during recognition of real objects (Fan et al., 2018), we might expect memorability to be a predictor of drawings’ recognizability. Additionally, the specific features that make a concept memorable might be the same features sketchers implicitly choose to include in their drawings of the concept.

To test this hypothesis, we correlated each object’s semantic neighbor preference with memorability scores for each of the THINGS objects collected by Kramer et al. (2023). These scores were measured in a behavioral experiment utilizing a continuous recognition memory task, where participants viewed a stream of images from the THINGS database and had to indicate with a button press whenever they remembered a repeated image from earlier in the stream. This resulted in over 1 million human memory ratings. The memorability for each image was then computed as the difference between the hit rate (HR), the proportion of correct identifications of repeats, and false-alarm rate (FAR), the proportion of incorrect detections. This resulted in a corrected recognition score, which has been shown to be highly similar across participants – people tended to consistently remember and forget the same images (Kramer et al., 2023).

We found no significant correlation between memorability and semantic neighbor preference (*r*(1852) = -0.028, *p*=0.21). Thus, while memorability may relate to semantic properties of visual concepts as captured by photographs (Kramer et al., 2023; Hovhannisyan et al., 2021), it does not account for people’s ability (or inability) to recognize abstract sketches of these concepts (Fig. 7 B).

Typicality is a salient property of members of a category that influences how quickly and easily the members are named (Rosch & Mervis, 1975; Rosch, 1975), how early they are acquired in development (Mervis & Pani, 1980), and how susceptible they are to miscategorization (McCloskey & Glucksberg, 1978). While one can judge how typical a given image is of a category, it is also possible to judge how typical a given category is of a higher-order superordinate category. Concepts that are generally more typical might support easier retrieval and facilitate recognition. We hypothesized that concepts that were more typical might be more easily accessible and thus might be both easier to draw and easier to recognize in sketches. We leveraged a set of typicality ratings for the THINGS concepts developed by Stoinski et al. (2023), and correlated SNP scores with typicality scores. Briefly, the typicality scores in Stoinski et al. (2023) were collected by asking participants to rank the typicality of a random subset of THINGS concepts with respect to a common parent category. For example, participants were shown concept words such as “dog”, “parrot”, “zebra” etc. and asked to rank them in terms of how representative they were of the category ‘animal’. The final typicality score for each concept reflected how often that concept was ranked highly in the subset in which it was presented. Concepts that were more typical of their superordinate categories had scores closer to 1 and atypical concepts were closer to 0. We found a moderately positive correlation between semantic neighbor preference and typicality (*r*(1852) = 0.35, $p <0.001$), supporting the notion that drawings of concepts that were more typical tended to contain visual features that led to semantically meaningful guesses from observers (Fig. 7 C).

## General discussion

Here, we introduced **DoT** , a large-scale dataset of over 28,000 drawings of 1854 visual object concepts representative of the human visual experience inherited from the THINGS database (Hebart et al., 2019, 2023). We focused on collecting high-quality drawings that nevertheless reflected the strategies and skill level of the average person without artistic training. We also collected dense multilabel recognition data on each drawing, allowing for rich comparisons with existing metadata on the THINGS concepts. This dataset builds on the tradition of using drawings to probe visual semantic knowledge (Fan et al., 2018; Yang & Fan, 2021; Mukherjee et al., 2019; Sangkloy et al., 2016; Eitz et al., 2012; Bainbridge et al., 2019), while addressing a key gap in existing datasets - semantic diversity. To demonstrate the utility of our dataset, we characterize the range of behaviors covered by our production and recognition experiment and present several analyses relating recognition performance to other aspects of cognition.

First, we note substantial variability in the recognizability of concepts when rendered as drawings, with some concepts being consistently identified correctly while others were never labeled accurately. Highlighting the interoperability of our dataset with sister datasets, we use recognition data on photographs of the THINGS concepts to show that there exist cases where the recognizability of drawings of concepts diverges from that of photographs of the same concepts. Interestingly, there were some concepts (11.38% of the database) where drawings were better recognized on average than their photographic counterparts, highlighting the potential of abstract visual representations to capture and communicate the diagnostic and essential features of visual object concepts that may not be as salient in real-world photographs. In contrast, cases where drawings fail to communicate the target concept might help delineate the limitations of hand-drawn renderings for communication in the absence of additional shared context. However, more generally, concepts that were difficult to recognize as drawings tended to also be challenging to identify in photographs, suggesting alignment in the features that make objects recognizable across different visual representations (Singer et al., 2023).

Our analysis of label diversity and semantic similarity provides evidence that even when participants do not provide the exact correct label for a drawing, their guesses tend to be semantically related to the true concept. This is reflected in the high semantic neighbor preference (SNP) scores observed for most concepts. Thus, even in cases where drawings fail to perfectly capture the target label, observers assign labels in a manner that is systematic and in the correct semantic neighborhood. While concept memorability did not significantly predict recognition performance, a concept’s typicality with respect to superordinate categories showed a moderate positive correlation with semantic neighbor preference. This indicates that more typical exemplars of a category may be easier to both draw and recognize, possibly due to their more accessible and well-defined features in semantic memory. Drawings allow for capturing high-fidelity information about people’s visual semantic knowledge that is impossible to capture using traditional measures. The use of drawings as the medium of production and as stimuli for a recognition study helped jointly show that people’s ability to visually communicate is highly varied and that this variation can potentially be explained as a function of concept-level properties.

The impetus behind the early standardized drawing datasets, such as the dataset by Snodgrass et al. (1980), was in standardizing the set of drawings being used in psychological experiments, thus allowing for generalizable findings across different experiments that utilized them. While careful attempts were made to control various aspects of the 280 drawings, including concept selection, drawing realism, and amount of detail, this approach did not capture how people naturally choose to depict these concepts. Large-scale datasets like QuickDraw solve this issue by collecting behavioral drawing data from people on the web, but run into the opposite problem of not being systematic in its selection of concepts. Here, in **DoT** , we have attempted to strike a balance by collecting a large-scale dataset from a standard crowdworking platform, while keeping our dataset couched in THINGS, a theoretically motivated and empirically validated database. Despite these considerable advantages, there are some limitations to our approach worth noting. It has been observed that there are marked effects of expertise and practice on drawing ability and related visuospatial skills (Chamberlain et al., 2021; Schlegel et al., 2015; Drake et al., 2024), and variance in expertise might lead to variability in the recognizability of drawings not explained by visual semantic knowledge. At the same time, a growing body of work has emphasized the importance of sequential pattern generation as a means of both measuring and inducing human concept learning (Lake et al., 2015; Ellis et al., 2015). **DoT** provides a data-rich testbed to evaluate these hypotheses at scale for a class of stimuli that are inherently semantic. Further, recent findings show that the recognizability of even children’s drawings is explained by semantic development independent of motor control (Long et al., 2024), signaling that limitations in participants’ drawing ability do not preclude measurement of their visual semantic knowledge using drawings. In fact, drawings of the majority of objects in **DoT** were recognizable above chance with many ‘incorrect’ labels resulting from observers assigning semantically neighboring object labels to drawings, and the majority of sketchers (> 98%) produced drawings that were, on average, recognizable above chance.

The items in **DoT** are drawings made under the specific task instructions of drawing from semantic memory (Kumar, 2021; Quillian, 1968), a task often referred to as simply ‘drawing’. Alternative modes of drawing production under different task contexts, such as delayed recall (Bainbridge et al., 2019; Patterson et al., 2007; Bozeat et al., 2003), copying (Sheppard et al., 2005; Gowen & Miall, 2007; Tchalenko & Miall, 2009; Perdreau & Cavanagh, 2015), and tracing (Cohen et al., 2021; Long et al., 2024; Gowen & Miall, 2007) often use the same general approach of allowing participants to create drawings but under more stringent goals and task conditions. These approaches have proven particularly useful in targeted investigations of cognitive processes in clinical populations. For example, recall-based drawing tasks have been used to characterize limitations in object memory in aphantasia (Bainbridge et al., 2021), hemispheric damage-related visuospatial neglect (Agrell & Dehlin, 1998; Cantagallo & Della Sala, 1998; Chen & Goedert, 2012), and knowledge degradation in semantic dementia (Patterson et al., 2007). While these different tasks confer their own unique benefits, the open-ended reference-free approach adopted in the present work allowed sketchers to include the visual features they thought most salient or representative of the object category without additional task demands. While we find a muted effect of memorability in predicting drawing recognizability in the present work, the memorability of a specific reference object in a copying or delayed recall task might be influential in predicting the recognizability of the resulting drawing. Future work can seek to explore the effect of different drawing paradigms on the constructs explored in this work.

Lastly, we note that drawing production is only one of many tools used to measure and characterize people’s knowledge of object concepts, albeit one that is complementary to standard psychophysical techniques, such as reaction times and recognition accuracies in response to naturalistic images (DiCarlo et al., 2012; Mack & Palmeri, 2015; Delorme et al., 2010), and neuroimaging measures in response to both drawings and images (Singer et al., 2023; Ishai et al., 2000). Drawings raise their own unique measurement problems regarding how to quantify the visual content within them, often relying on pre-trained deep neural network feature extractors (Fan et al., 2018; Singer et al., 2022; Mukherjee et al., 2024; Baker et al., 2018) or human annotations (Mukherjee et al., 2019; Bainbridge et al., 2019; Huey et al., 2023; Chen & Goedert, 2012; Chamberlain et al., 2015; Sayim & Wagemans, 2017), potentially making any inferences derived from drawings more susceptible to noise. Thus, a wide variety of measures, including drawing, should be brought to bear when characterizing human visual concept knowledge.

While we present our analyses as a starting point, the **DoT** dataset opens up several avenues for future research, including further fine-grained investigations into human semantic memory, the faculties that support visual abstraction, and the computational principles that support sketch recognition and generation, potentially leading to better models of how humans represent and communicate visual concepts. In conclusion, drawings of THINGS provides a rich resource for investigating the structure and organization of visual semantic knowledge. By combining a diverse set of object concepts with both production and recognition data, this dataset enables researchers to ask nuanced questions about how humans represent, communicate, and interpret visual information.

## Supplementary Information

Below is the link to the electronic supplementary material.

Supplementary file 1 (pdf 2758 KB)

## Data Availability

The experiments reported in this manuscript were not preregistered. Data for the experiments reported can be accessed at the following OSF repository: https://osf.io/s9yta/ All pre-processing and analyses reported are available at: github.com/cogtoolslab/drawings-of-THINGS-public

## Footnotes

1 Of the 1314 participants in the drawing production study, 50 did not complete all trials, including the demographic information trial. Our gender and age breakdowns do not include data from these participants.

2 Of the 1557 participants in the recognition study, 101 did not complete all trials, including the demographic information trial. Our gender and age breakdowns do not include data from these participants.

3 We report recognizability both based on the first guess (top-1 guess) or the best guess amongst all labels entered by a participant (top-k guess).

4 This *p* value was derived using a permutation test where the image-to-drawing similarity matrix was shuffled 1000 times to obtain a null distribution of the difference between the within-category and between-category means.

5 Confidence Intervals were computed using the standard deviation of the difference scores across concepts. This is because difference scores for individual objects were derived by computing the difference of recognizability scores between drawings and images and thus had no variance measure.
