# Supplementary material for: Drawings of THINGS: A large-scale drawing dataset of 1854 object concepts
Source: Behav Res Methods. 2026 Jan 30;58(2):57. doi: 10.3758/s13428-025-02887-w (PMC12858628; doi:10.3758/s13428-025-02887-w)
Supplement: Supplementary file 1 — (pdf 2758 KB) [file 13428_2025_2887_MOESM1_ESM.pdf]

Supplementary Materials

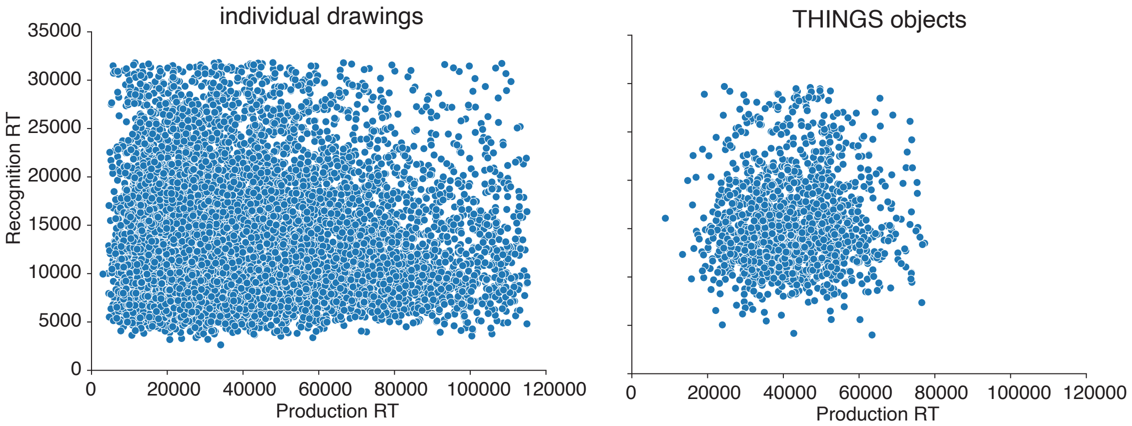

Figure S1

Relationship between drawing production and recognition response times. The left figure shows the time taken to produce vs. the time taken to complete providing all labels on average for each drawing in **DoT***. Only drawings recognized correctly were considered for this analysis. Outlier drawings with RTs greater than 1.5 times the inter-quartile range of observed RTs were removed. We observed a weak but statistically significant positive correlation ($r = 0.04$; $p < 0.001$). The figure on the right shows the same relationship but after averaging production and recognition response times at the object level. Here too, we found a weak but significant positive correlation ($r = 0.06$; $p < 0.001$)*

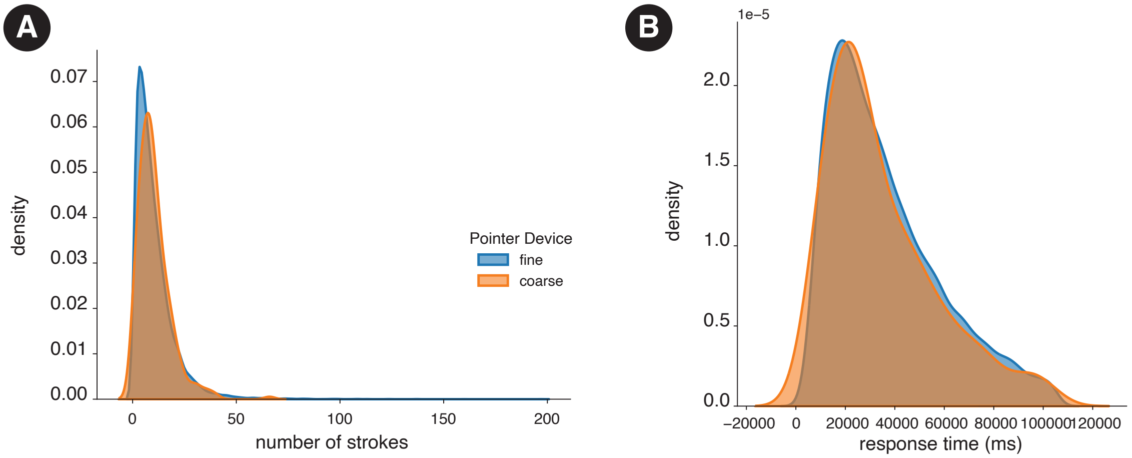

Figure S2

(A) Distribution of number of strokes used to make the drawings in **`DoT`** *separated by whether the drawing 'pointer' device logged by the participant's browser was classified as 'fine' or 'coarse'. There was no significant difference between the two device types in terms of number of strokes used ($t = 0.26$, $p = 0.79$) (B) Distribution of drawing response times separated by drawing device type. Here too, there was no significant difference between the two device types in terms of the amount of time taken to complete drawings ($t = 1.35$, $p = 0.18$). Thus, in general, the information about drawing devices provided by participants' browsers were not predicting of level of detail (number of strokes) or the amount of time taken to make a drawing (RT).*
